# Investigation into the role of the cholinergic system in radiation-induced damage in the rat liver and ileum

**DOI:** 10.1093/jrr/rru039

**Published:** 2014-06-08

**Authors:** Hazan Özyurt, A. Sevgi Özden, Özge Çevİk, Zerrin Özgen, Selin ÇadIrcI, Merve Açıkel Elmas, Feriha Ercan, Göksel Şener, M.Z. Gören

**Affiliations:** 1 Dr Lutfi Kirdar Kartal Training and Research Hospital, Radiation Oncology, 34865 Istanbul, Turkey; 2 Cumhuriyet University School of Pharmacy, Department of Biochemistry, 58140 Sivas, Turkey; 3 Marmara University Pendik Training and Research Hospital, Radiation Oncology, Üst Kaynarca, Istanbul, Turkey; 4 Marmara University School of Pharmacy, Pharmacology, 34668 Istanbul, Turkey; 5 Marmara University School of Medicine, Department of Histology and Embryology, Başıbüyük Health Campus, Basic Medical Sciences Building, Başıbüyük, Maltepe, 34854 Istanbul, Turkey; 6 Marmara University School of Medicine, Department of Medical Pharmacology, Başıbüyük Health Campus, Basic Medical Sciences Building, Başıbüyük, Maltepe, 34854 Istanbul, Turkey

**Keywords:** IL-1*β*, IL-10, TNF-*α*, myeloperoxidase activity, caspase-3

## Abstract

It has been previously shown that acetylcholine (ACh) may affect pro-inflammatory and anti-inflammatory cytokines. The role of the cholinergic system in radiation-induced inflammatory responses and tissue damage remains unclear. Therefore, the present study was designed to determine the radio-protective properties of the cholinergic system in the ileum and the liver of rats. Rats were exposed to 8-Gy single-fraction whole-abdominal irradiation and were then decapitated at either 36 h or 10 d post-irradiation. The rats were treated either with intraperitoneal physiological saline (1 ml/kg), physostigmine (80 µg/kg) or atropine (50 μg/kg) twice daily for 36 h or 10 d. Cardiac blood samples and liver and ileal tissues were obtained in which TNF-*α*, IL-1*β* and IL-10 levels were assayed using ELISA. In the liver and ileal homogenates, caspase-3 immunoblots were performed and myeloperoxidase (MPO) activity was analyzed. Plasma levels of IL-1*β* and TNF-*α* increased significantly following radiation (*P* < 0.01 and *P* < 0.001, respectively) as compared with non-irradiated controls, and physostigmine treatment prevented the increase in the pro-inflammatory cytokines (*P* < 0.01 and *P* < 0.001, respectively). Plasma IL-10 levels were not found to be significantly changed following radiation, whereas physostigmine augmented IL-10 levels during the late phase (*P* *<* 0.01). In the liver and ileum homogenates, IL-1*β* and TNF-*α* levels were also elevated following radiation, and this effect was inhibited by physostigmine treatment but not by atropine. Similarly, physostigmine also reversed the changes in MPO activity and in the caspase-3 levels in the liver and ileum. Histological examination revealed related changes. Physostigmine experiments suggested that ACh has a radio-protective effect not involving the muscarinic receptors.

## INTRODUCTION

Inflammation is a response to altered homeostasis due to an infection, a trauma or other noxious stimuli, and it is characterized by the release of many pro-inflammatory substances, vasoactive mediators and free radicals [1]. Acute phase cytokines can be divided into three groups. The first group members are the pro-inflammatory cytokines that initiate or enhance the cascade of events, and Tumor Necrosis Factor-alpha (TNF-*α*), interleukin-1 (IL-1), interferon-gamma (IFN-*γ*) and interleukin-8 (IL-8) are important pro-inflammatory cytokines. Interleukin-6 (IL-6), leukemia inhibitory factor (LIF), interleukin-11 (IL-11), oncostatin M, ciliary neurotrophic factor and cardiotrophin-1 are acute phase cytokines involved in the tissues. The third group consists of anti-inflammatory cytokines that downregulate the acute phase response substances, such as interleukin-10 (IL-10), interleukin-4 (IL-4), interleukin-13 (IL-13) and transforming growth factor-beta [2]. Studies performed on acetylcholine (ACh) release and/or on the non-neuronal cholinergic system have demonstrated that both endothelial cells and T lymphocytes produce ACh [3–5]. ACh also inhibits the lipopolysaccharide-induced production of pro-inflammatory cytokines (including IL-1) from macrophages and microglia [6–8]. Acute radiation can produce damage in tissues having rapidly proliferating cells, such as the skin or alimentary tract [9]. Radiation injury can produce acute or chronic toxicities. Acute toxicity occurs during and shortly after the radiotherapy and is characterized by symptoms such as diarrhea, abdominal pain, nausea, vomiting, bleeding, increased stool frequency, rectal–anal pain, decreased food uptake, and fluid and electrolyte loss [9]. Chronic intestinal radiation injury occurs at least 3 months after the treatment and causes a range of symptoms, such as a change in bowel pattern, diarrhea, fecal incontinence, pain, and intestinal blood loss [9]. Following irradiation to the abdomen, radiation-induced liver disease can occur in patients with normal liver function, causing anicteric hepatomegaly and mild alkaline phosphatase serum level elevation. It has also been reported that a more severe derangement of liver function occurs in patients with pre-existing liver disease, and radiation-induced liver disease can progress to fibrosis, cirrhosis, and liver failure [10].

Myeloperoxidase (MPO) is the most abundant pro-inflammatory enzyme stored in the azurophilic granules of neutrophils, and it catalyzes the formation of hypochlorous acid from hydrogen peroxide and chloride ions during respiratory burst of the neutrophils. It generates other highly reactive molecules (such as tyrosyl radicals) and cross-links proteins. It has also been reported that intracellular MPO activity correlates well with tissue neutrophil content and serves as a marker to assess neutrophil infiltration in a tissue [11]. Traditionally, apoptosis has been considered to be the predominant type of programmed cell death and the major form of cell death induced by ionizing radiation. Combined inhibition of apoptosis and of the mammalian target or rapamycin pathway (mTOR) during radiotherapy is a potential therapeutic strategy for enhancing radiation therapy for patients with non-small-cell lung cancer [12]. ACh is an important neurotransmitter that acts on two types of cholinergic receptors: nicotinic and muscarinic receptors [13]. Atropine, a belladonna alkaloid, specifically antagonizes muscarinic-type cholinergic receptors [13]. ACh is degraded by cholinesterases and has a very short half-life, and this property makes it unsuitable for being used as a drug [13]. Instead, cholinesterase inhibitors (such as physostigmine) are used to mimic the effects of ACh and hence act as agonists [13].

This study sought to demonstrate a possible role for the cholinergic system by using physostigmine or atropine on inflammation present 36 h and 10 d after abdominal radiation in rats. The rationale was to investigate a potential role for cholinergic agents as new adjuvants for preventing tissue damage during irradiation. For this reason, TNF-*α*, IL-1*β* and IL-10 levels in plasma samples, and caspase 3 and MPO activities were investigated in liver and ileal homogenates, and histological slides were evaluated.

## MATERIALS AND METHODS

### Animal ethics

A total of 49 Sprague Dawley rats (250–300 g) supplied by Marmara University Animal Center (DEHAMER) were housed in an air-conditioned room with 12:12 light/dark cycles where the temperature (22 ± 2°C) and relative humidity (65–70%) were kept constant. The rats were fed with standard rat chow and water *ad libitum*. All of the experimental protocols were approved by the University Animal Care and Use Committee. Each group was comprised of seven rats.

### Experimental protocols

Anesthetized rats were placed on a Styrofoam block side by side in a supine position, and paraffin was used to fill the gaps between the rats. The limbs were extended and stabilized with plaster. Single-fraction 8-Gy whole-abdominal irradiation was implemented in a linear accelerator (Clinac DHX, Varian Medical Systems Inc., USA) with 6-MV X-rays at a source-to-surface distance of 100 cm. The rats were assigned to early (36 h) or late (10 d) post-irradiation phase groups. The irradiation protocol was applied 30 min following the first treatment. Upon completion of the irradiation procedure, the rats received intraperitoneal physiological saline (1 ml/kg), physostigmine (80 μg/kg) or atropine (50 μg/kg) every 12 h. The rats in the early-phase group were sacrificed 36 h after irradiation. The late-phase group rats continued to receive the treatments for 10 d and were eventually sacrificed upon completion of the protocol. The doses of physostigmine [14–17] and atropine [18] used in this study were based on previous studies. The rats were anesthetized with ether, and cardiac blood samples were taken. The rats were then decapitated, and the pro- and anti-inflammatory cytokines were assayed in the plasma and liver and ileal tissues. Caspase levels and MPO activity were also analyzed in the liver and ileal tissues, and morphological evaluation of the tissues was performed. Naïve control rats were used for the analyses of the basal parameters.

### Blood assays

Plasma and tissue levels of IL-10, IL-1*β* and TNF-*α* were quantified using enzyme-linked immunosorbent assay (ELISA) kits specific for the previously mentioned rat cytokines according to the manufacturer's instructions and guidelines (Biosource Europe SA, Nivelles, Belgium).

### Tissue MPO activity determination

The MPO activity was measured in tissues using a procedure similar to that documented by Hillegas *et al.* [19]. Tissue samples were homogenized in 10 volumes of ice-cold potassium buffer (20 mM KH_2_PO_4_, pH 7.4) and then centrifuged at 40 000 g for 10 min at 4°C. The pellet was then rehomogenized with an equivalent volume of 50 mM KH_2_PO_4_ containing 0.5% (w/v) hexadecyltrimethylammonium bromide. MPO activity was assessed by measuring the H_2_O_2_-dependent oxidation of o-dianizidine 2 HCl. One unit of enzyme activity was defined as the amount of MPO present that caused a change in absorbance of 1.0/min at 460 nm and 37°C. The results were expressed as U/g wet tissue.

### Measurement of tissue caspase-3 activity

The caspase-3 activity assay was performed using the caspase-3 cellular activity assay kit (Calbiochem, San Diego, CA) according to the manufacturer's instructions. Tissue samples were treated for 10 min with iced lysis buffer supplied by the manufacturers. Then 40 µl of tissue samples and 50 µl assay buffer (100 mM NaCl, 50 mM HEPES, 10 mM DTT, 1 mM EDTA, 10% glycerol, 0.1% CHAPS, pH 7.4) were added to wells, and the microplate was equilibrated at 37°C for 10 min. The reaction was initiated by adding 10 µl of DEVD-pNA substrate (200 mM final concentration). The colorimetric release of *p*-nitroaniline (pNA) from the Ac-DEVD-pNA substrate was recorded from 0 to 60 min at 405 nm using the specific activity of DEVD-pNa cleavage (pmol pNA/min) for each sample. It was calculated by measuring the slope of the linear portion of the absorbance vs time graph, as follows: specific activity = (slope [(sample/min)] × ([50 mM/A_405_ (100 µl of 50 mM pNA)]) × 100 µl (assay volume). The DEVD-pNA cleavage activity was calculated in pmol/min/mg protein. Protein concentration in tissue samples was determined using the Bradford method [20].

### Histological evaluation

For the light microscopic investigations, liver and ileum samples were ﬁxed with 10% formaldehyde, dehydrated in a graded alcohol series, cleared in toluene and embedded in parafﬁn. Tissue sections (5 μm) were stained with hematoxylin and eosin and examined under an Olympus BX51 photomicroscope (Tokyo, Japan). All tissue sections were examined microscopically for the characterization of histopathological changes by an experienced histologist who was unaware of the treatment conditions.

### Statistical analysis

The results were expressed as mean ± SEM. One-way analysis of variance followed by Tukey's Multiple Comparison test as a *post hoc* test was used for statistical analysis, where *P* < 0.05 was the accepted level for statistical significance.

## RESULTS

### The effect of radiation and cholinergic agents on the plasma levels of IL-10, IL-1*β* and TNF-*α* in rats

**Fig. 1.**
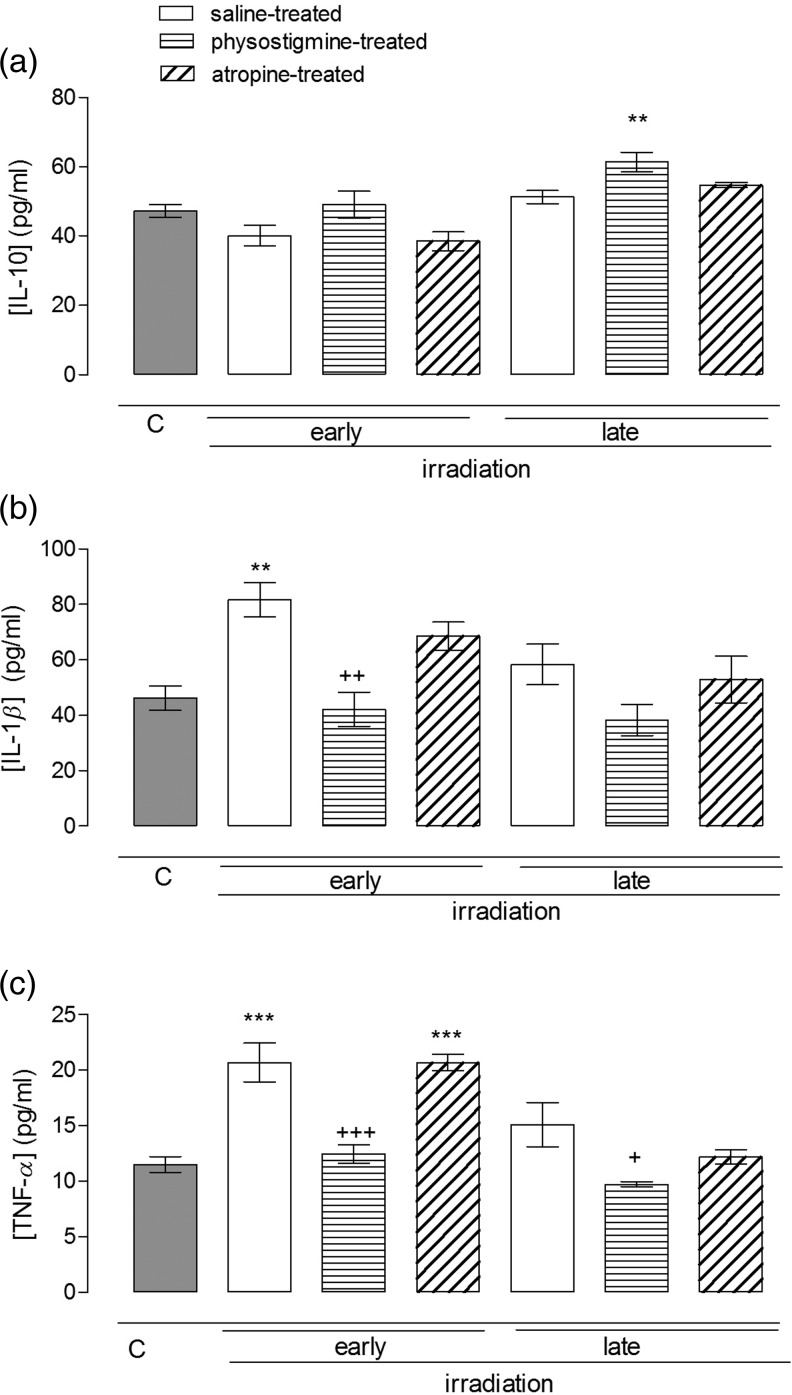
Plasma IL-10 (**a**), TNF-*α* (**b**) and IL-1*β* levels (**c**) in the early (36 h) and late (10 d) phases of non-irradiated controls and saline-, physostigmine- and atropine-treated irradiated groups (*n* = 7 rats/group). **P < 0.01, ***P < 0.001: vs control group. ^+^*P* < 0.05, ^++^*P* < 0.01, ^+++^*P* < 0.001: vs saline-treated irradiated group.

IL-10 levels in the plasma samples of rats treated with physiological saline collected 36 h after irradiation were not found to be different from those of controls (Fig. 1a). Similarly, in the early period neither physostigmine nor atropine treatments affected the IL-10 levels in the irradiated rats. The IL-10 levels collected from rats 10 d after irradiation treated with physostigmine, however, were observed to be significantly higher than those of control rats (*P* *<* 0.05; Fig. 1a).

IL-1*β* levels increased from 46.25 ± 4.47 pg/ml to 81.70 ± 6.06 pg/ml within 36 h, and statistical comparison yielded a significant difference (*P* *<* 0.01; Fig. 1b). In the early period, the ACh esterase inhibitor physostigmine significantly reduced the increases in IL-1*β* levels produced by irradiation (*P* *<* 0.01). However, atropine treatment produced no significant effect on IL-1*β* levels in the early period. No significant change was observed in IL-1*β* levels during the late period.

Analysis of TNF-*α* levels revealed that radiation produced an increase in the saline-treated rats (*P* *<* 0.001; Fig. 1c) that was reduced by physostigmine (*P* *<* 0.001). Physostigmine treatment also dampened the increase in TNF-*α* levels in the late period. Atropine produced no significant effect on the TNF-*α* levels in either early or late phases.

### The effect of radiation and cholinergic agents on IL-10, IL-1*β* and TNF-*α* levels measured in the liver homogenates of rats

**Fig. 2.**
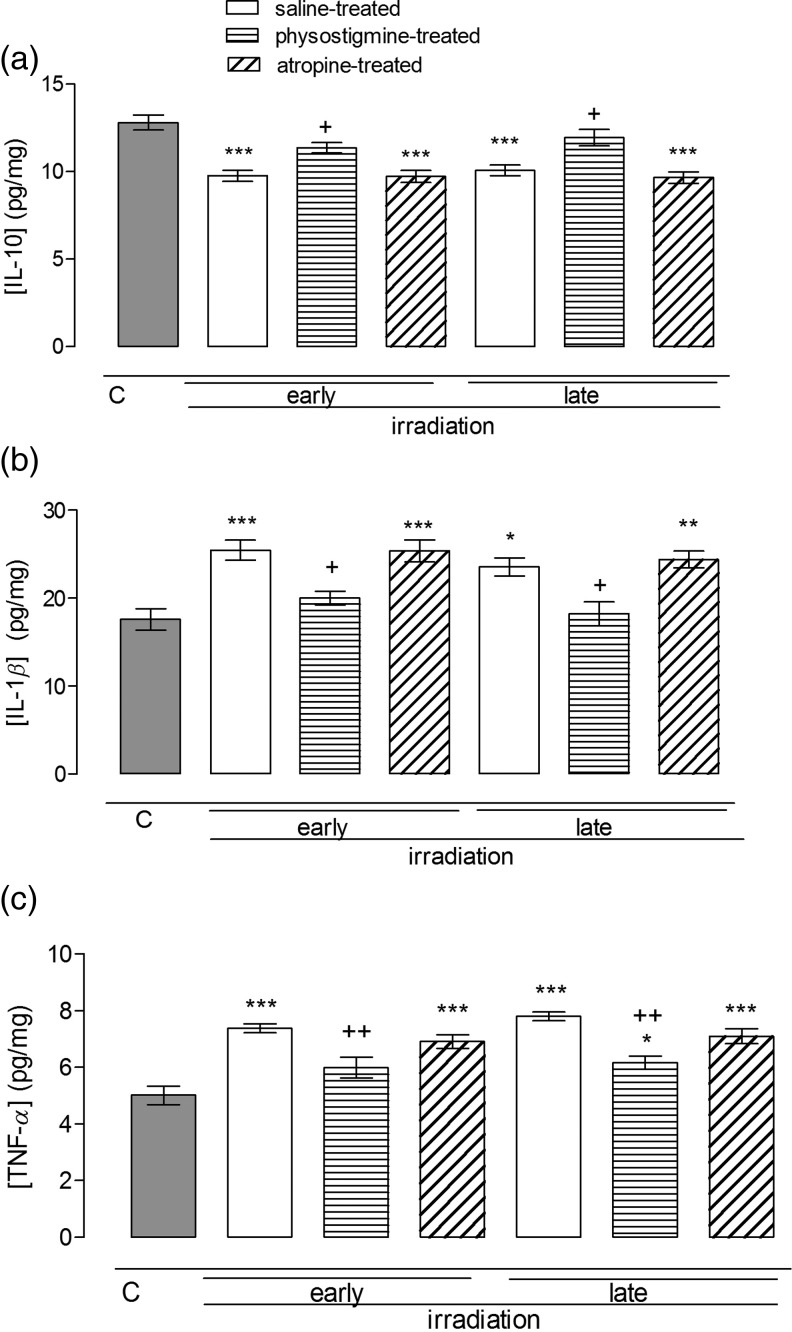
Hepatic tissue IL-10 (**a**), TNF-*α* (**b**) and IL-1*β* (**c**) levels in the early (36 h) and late (10 d) phases of non-irradiated controls and saline-, physostigmine- or atropine-treated irradiated groups (*n* = 7 rats/group). *P < 0.05, **P < 0.01, ***P < 0.001: vs control group. ^+^*P* < 0.05, ^++^*P* < 0.01: vs saline-treated irradiated group.

In the liver homogenates, IL-10 levels decreased in irradiated controls significantly compared with those of control rats both in the early- and the late-period groups (*P* *<* 0.001; Fig. 2a). Physostigmine treatment reversed the decreases as compared with irradiated controls both in the early- and late-period groups (*P* *<* 0.05; Fig. 2a). Atropine-treated groups were found to be similar to the irradiated controls, indicating that atropine produced no effect. IL-1*β* and TNF-*α* levels displayed similar changes, i.e. in the irradiated groups, IL-1*β* and TNF-*α* levels increased (*P* *<* 0.001) but were restored to basal levels by physostigmine treatments (*P* *<* 0.05 and *P* *<* 0.01, respectively; Fig. 2b and c), and atropine treatment failed to affect IL-1*β* and TNF-*α* levels, either in the early or the late periods.

### The effect of radiation and cholinergic agents on IL-10, IL-1*β* and TNF-*α* levels measured in the rat ileal homogenates

**Fig. 3.**
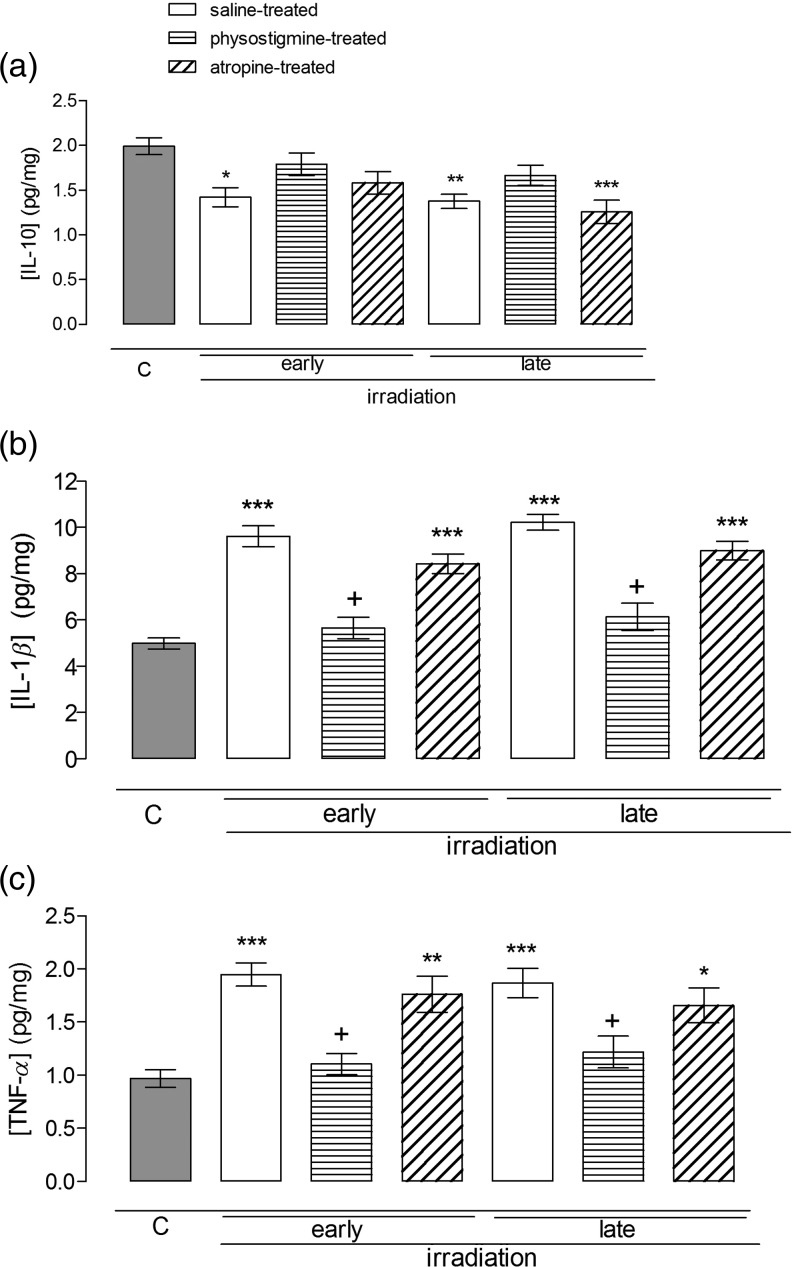
Ileal tissue IL-10 (**a**), TNF-*α* (**b**) and IL-1*β* (**c**) levels in the early (36 h) and late (10 d) phases of non-irradiated controls and saline-, physostigmine- or atropine-treated irradiated groups (*n* = 7 rats/group). *P < 0.05, **P < 0.01, ***P < 0.001: vs control group. ^+^*P* < 0.05, ^++^*P* < 0.001: vs saline-treated irradiated group.

In the ileal homogenates, IL-10 levels decreased in irradiated controls significantly as compared with control rats both in the early- and the late-period groups (*P* *<* 0.05 and *P* *<* 0.01, respectively; Fig. 3a). Physostigmine produced IL-10 levels in the irradiated control group similar to those of non-irradiated rats (Fig. 3a). Atropine-treated rats maintained lower IL-10 levels in the late group (*P* *<* 0.001; Fig. 3a). IL-1*β* and TNF-*α* levels were increased in the irradiated controls (*P* *<* 0.001; Fig. 3b and c), and these increases were offset by physostigmine treatments (*P* *<* 0.05; Fig. 3b and c); atropine-treated groups displayed similar patterns to those of the irradiated controls, being significantly higher than the non-irradiated control groups (*P* *<* 0.05; Fig. 3b and c).

### The effect of radiation and cholinergic agents on MPO activity and caspase 3 levels measured in the rat ileal and liver homogenates

**Fig. 4.**
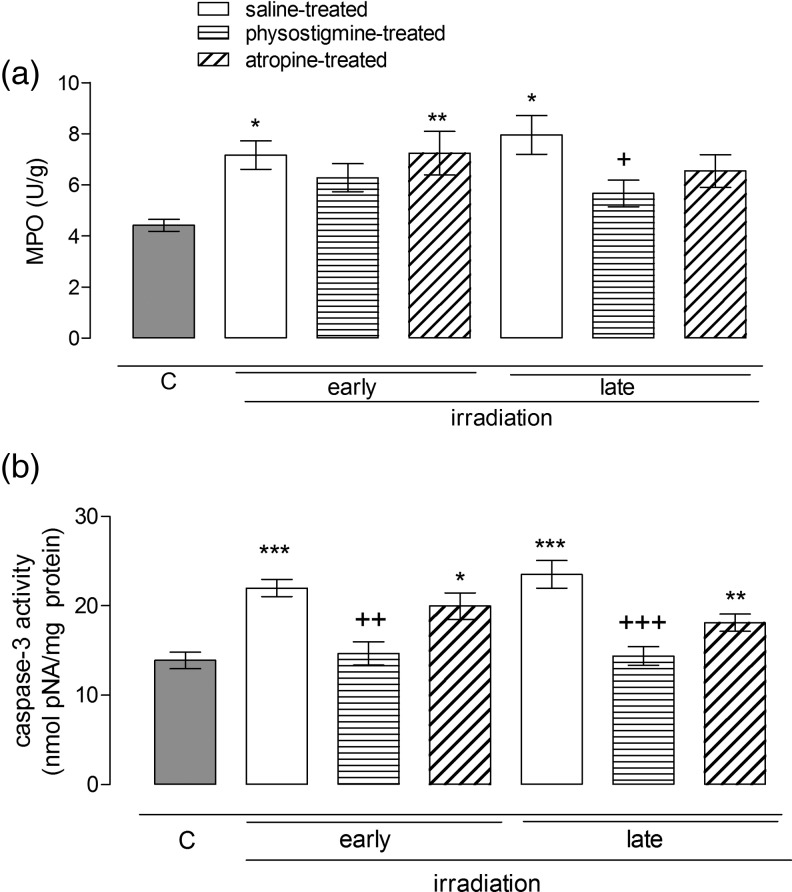
Hepatic tissue myeloperoxidase (**a**) and caspase-3 (**b**) activities in the early (36 h) and late (10 d) period of control and saline-, physostigmine- or atropine-treated irradiated groups (*n* = 7 rats/group). *P < 0.05, **P < 0.01, ***P < 0.001: vs control group. ^+^*P* < 0.05, ^++^*P* < 0.01, ^+++^*P* < 0.001: vs saline-treated irradiated group.

**Fig. 5.**
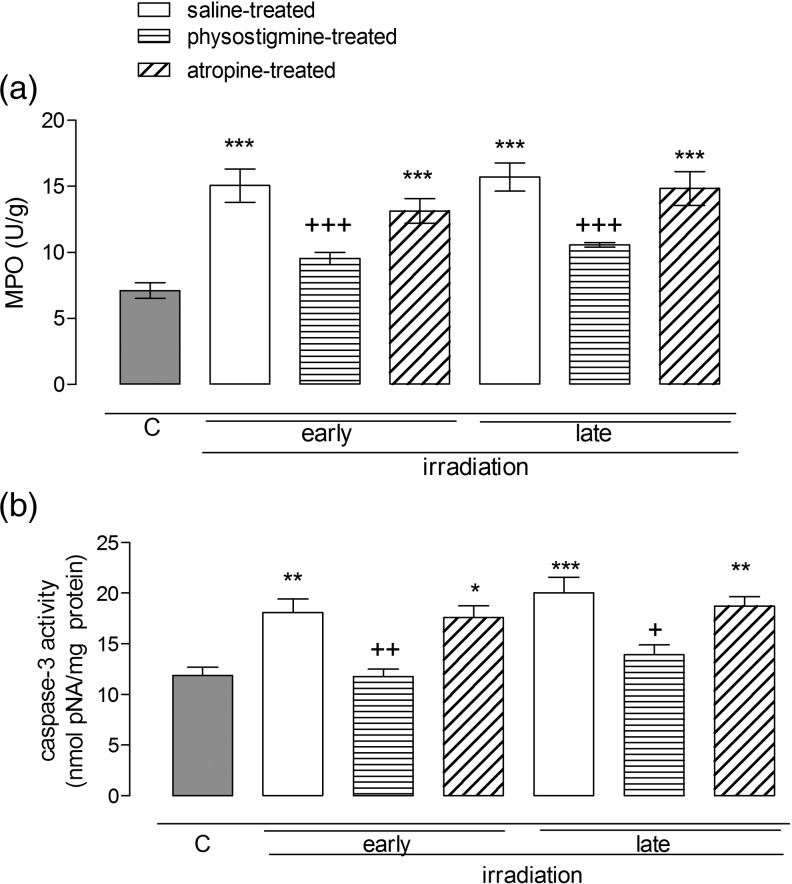
Ileal tissue myeloperoxidase (**a**) and caspase-3 (**b**) activities in the early (36 h) and late (10 d) period of control and saline-, physostigmine- or atropine-treated irradiated groups (*n* = 7 rats/group). *P < 0.05, **P < 0.01, ***P < 0.001: vs control group. ^+^*P* < 0.05, ^++^*P* < 0.01, ^+++^*P* < 0.001: vs saline-treated irradiated group.

Radiation increased MPO activity in the liver homogenates both in the early and late phases following radiation compared with that in controls (Fig. 4a; *P* *<* 0.05). The physostigmine-treated group was not found to differ from the non-irradiated controls, indicating that physostigmine treatment became effective in both phases. However, atropine-treated rats also had higher MPO activity in the early phase. Likewise, caspase-3 levels were also significantly higher than those of controls in the irradiated rats, both in the early and late phases (Fig. 4b; *P* *<* 0.001), and physostigmine prevented these increments (*P* < 0.05). In the ileal homogenates, radiation significantly elevated MPO activity in the early and late phases (Fig. 5a; *P* *<* 0.001), whereas physostigmine-treated groups were found to be similar to controls (Fig. 5a; *P* *<* 0.001). Caspase-3 levels were also increased following irradiation, whereas physostigmine-treated groups displayed similar levels to those of controls and yielded non-significant *P* values; comparing the physostigmine-treated groups with the irradiated group yielded significant *P* values (*P* < 0.05) (Fig. 5b). Atropine-treated rats displayed similar MPO activities and caspase-3 levels to those of saline-treated irradiated rats, indicating that atropine is not effective in restoring these parameters.

### Histology

**Fig. 6.**
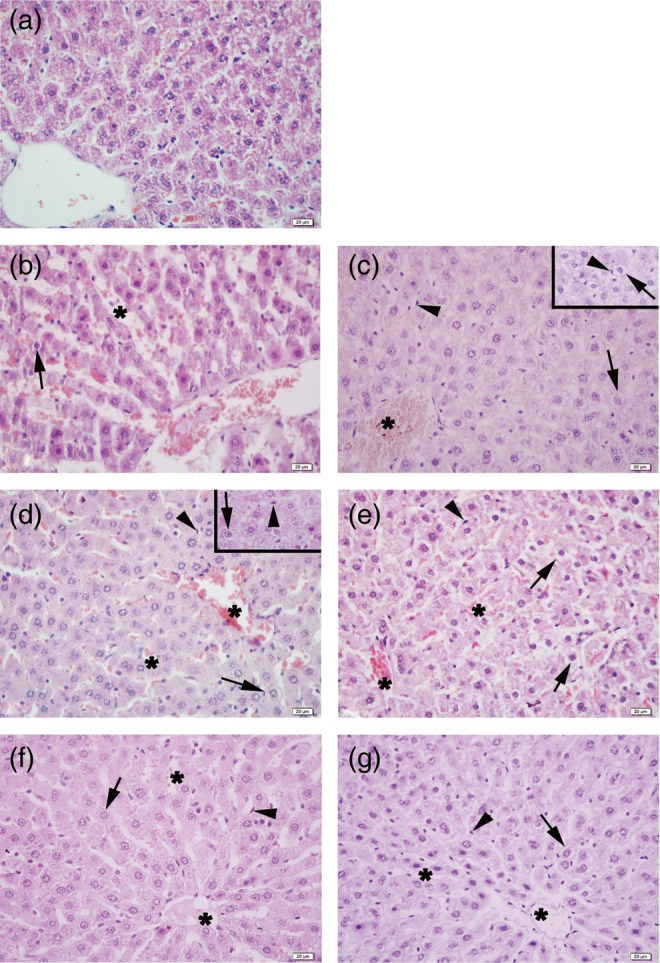
Representative micrographs of the liver tissue in the experimental groups. Regular hepatocyte parenchyma with sinusoids and hepatocytes in the control group (**a**). Severe sinusoidal congestion (stars), degenerated hepatocytes with perinuclear vacuolization (arrows) and activated Kupffer cells (arrowheads) in the irradiated early-phase (**b**) and the late period (**c**) of the irradiated group. Moderate sinusoidal congestion (stars), degenerated hepatocytes with perinuclear vacuolization (arrows) and activated Kupffer cells (arrowheads) in atropine-treated irradiated rats in early (**d**) and late (**e**) phases. Mild sinusoidal congestion (stars), a few degenerated hepatocytes (arrows) and activated Kupffer cells (arrowheads) in physostigmine-treated irradiated rats in early (**f**) and late (**g**) phases. H&E staining, scale bars: 20 µm.

Light microscopic evaluation of the control group revealed a regular morphology of liver parenchyma with intact hepatocytes and sinusoids. Severe sinusoidal congestion and hemorrhage, dilation of the central vein, degenerated hepatocytes with perinuclear vacuolization and activated Kupffer cells were observed in the irradiated groups both in the early and late phases. Atropine treatment produced moderate sinusoidal congestion, degenerated hepatocytes with perinuclear vacuolization and activated Kupffer cells in the early and late phases of irradiated groups, whereas physostigmine treatment produced well-preserved liver parenchyma with sinusoids, hepatocytes and Kupffer cells in both phases in irradiated rats (Fig. 6).

**Fig. 7.**
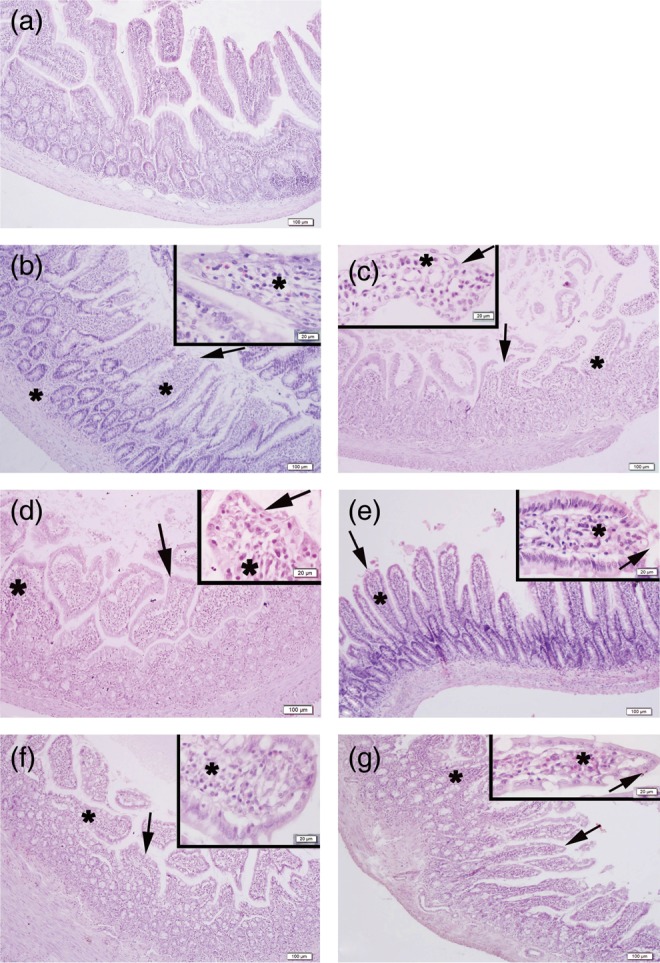
Representative micrographs of the ileal tissue in the experimental groups (**a**). Regular villi morphology with epithelium and lamina propria in the control group (**b**). Epithelial desquamation (arrows) and severe inflammatory cell infiltration (stars) in the irradiated rats in early (**b**) and late (**c**) phases. Regular surface epithelium (arrows) and moderate inflammation in atropine-treated irradiated rats in early (**d**) and late (**e**) phases. Regular surface epithelium (arrows) and mild inflammation in physostigmine-treated irradiated rats in early (**f**) and late (**g**) phases. H&E staining, scale bars: 20 µm, insets: 100 µm.

The light microscopy ﬁndings of the small-bowel mucosa were entirely normal in the control group. Severe degeneration of villi with epithelial desquamation and severe inflammatory cell infiltration were observed in both early- and late-phase irradiated groups. Atropine treatment produced mild degeneration in the surface epithelium and moderate inﬂammation, and physostigmine treatment produced mild degeneration in the surface epithelium and mild inﬂammation in the early and late phases in irradiated groups (Fig. 7).

## DISCUSSION

Radiotherapy is widely used for the treatment of solid organ malignancies in the abdomen and pelvis. The intestine is highly radiosensitive, and this property makes it a dose-limiting organ both in total body and abdominopelvic radiation procedures since radiation-induced enteritis (with a range of deleterious intestinal symptoms, including bleeding, anorexia, nausea, vomiting and diarrhea) can be a challenging clinical problem [21–23]. In this current study, we demonstrated that the levels of the pro-inflammatory cytokines IL-1*β* and TNF-*α* significantly increased in plasma in the early phase following radiotherapy, but no significant changes were observed in the late phase. In a previous study performed in mice, plasma TNF-*α* and IL-10 levels were shown to be elevated 6 h after abdominal radiation [24]. Collectively, in prostate cancer patients an immediate elevation of IL-1*β* and IL-6 in plasma following irradiation has also been demonstrated [25]. In that report, cytokine levels appeared to peak 1–2 weeks after radiotherapy. This may indicate that the timing of our protocol may be appropriate for human experiments. TNF-*α*, a primary and potent mediator of inflammation synthesized mainly by monocytes, macrophages and T cells, has a short half-life. It is also the initiator of the successive cytokine cascade and a potent inducer of other inflammatory cytokines [26].

IL-1 has been found in two different types to date, IL-1*α* and IL-1 *β*, and both evoke metabolic and hemodynamic changes similar to those of TNF-*α* and activate production of other cytokines [26–28]. In our study, we also demonstrated increased levels of the anti-inflammatory cytokine IL-10 during the late phase. This finding may indicate that the anti-inflammatory cytokines are secreted as a response to pro-inflammatory cytokines to maintain homeostasis. To avoid an inappropriate, excessive inflammatory response, a number of anti-inflammatory mechanisms exist to prevent inflammatory mediators entering the circulation. Anti-inflammatory cytokines such as IL-10, TNF-binding protein and transforming growth factor-*β* are produced during the normal immune response and can inhibit the release of TNF-*α* and other pro-inflammatory cytokines [29, 30].

Our investigations into the use of the ACh esterase inhibitor physostigmine demonstrated that it could suppress pro-inflammatory cytokines during the early phase. Furthermore, it also caused the anti-inflammatory cytokine IL-10 to be increased during the late phase. Macrophages and other cytokine-producing cells express ACh receptors, and studies indicate that ACh suppresses TNF-*α* synthesis and inhibits the release of IL-1*β*, IL-6 and IL-8 without preventing the release of the anti-inflammatory cytokine IL-10 [31]. There is a vast amount of literature describing the role of ACh in inflammation. Systemic anti-inflammatory responses to endotoxins are modulated by the activation of efferent vagus nerve fibers [32]. Vagus nerve-dependent release of ACh at pre-splenic synapses generates an anti-inflammatory effect by inhibiting the production of pro-inflammatory cytokines without affecting IL-10, the anti-inflammatory cytokine [32]. Peripheral blood mononuclear cells were found to express nicotinic and muscarinic ACh receptors [33–35]. Besides macrophages, other cells of the immune system express nicotinic and muscarinic receptors, and ACh released from lymphocytes or endothelial cells acts in an autocrine or paracrine way that modifies immune responses [31]. ACh acts on both nicotinic and muscarinic types of cholinergic receptors. In our study we could not demonstrate reversal of the responses by treatment with atropine, a non-specific muscarinic antagonist, however, the results of our experiments may suggest a role for nicotinic receptors during inflammatory responses following radiotherapy. Initial studies indicated that ACh acts on the *α*-bungarotoxin-sensitive nicotinic receptors on human macrophages [33]. Electrical stimulation of the vagus nerve was shown to inhibit macrophage TNF-α synthesis in wild-type mice but not in *α*7-deficient mice, thus demonstrating the *α*7nAChR to be an essential peripheral component of the cholinergic anti-inflammatory pathway [36].

The administration of ACh esterase inhibitors enhances cholinergic signaling and can suppress inflammation of the brain and periphery [37]. Galantamine, an ACh esterase inhibitor, attenuates serum TNF-*α* and improves survival in murine endotoxemia [38]. All these studies suggest that the cholinergic anti-inflammatory pathway does not utilize peripheral muscarinic signaling during inflammation. However, activation of muscarinic receptors was shown to exert pro-inflammatory effects in a wide variety of cells implicated in airway inflammation. Activation of M3 subtype receptors could stimulate bovine alveolar macrophages and human sputum macrophages to release leukotriene B4 and other mediators [39]. Anticholinergic drugs such as tiotropium bromide could regulate the release of chemotactic factors from human epithelial cells and macrophages *in vitro* [39]. It was also demonstrated that atropine produces a radio-protectant effect in radiation-induced crypt depletion and epithelial proliferative capacity [40]. Similarly, our experiments indicated a similar trend on the microscopic slides of ileum treated with atropine, although statistical analyses yielded no significant changes.

MPO is an enzyme that is found predominantly in the azurophilic granules of polymorphonuclear leukocytes. Radiation increased MPO activity both in ileum and liver homogenates collected during early and late phases. However physostigmine decreased MPO activity more profoundly in the ileum samples. Atropine again failed to affect radiation-induced MPO activity. A previous study showed that the intestinal MPO activity in irradiated rats was increased significantly, indicating that radiation-induced oxidative injury in this tissue involves the contribution of neutrophil accumulation radiotherapy [41]. It has been reported that irradiating biological material leads to a rapid burst of reactive oxygen metabolites such as superoxide, hydrogen peroxide and hydroxyl groups (**^.^**OH9) [42]. These metabolites interact with biological target molecules, causing lipid peroxidation and DNA damage, and subsequently cell killing and mutations occur [43]. In the current study, MPO activity was found to increase in the liver and ileum homogenates, indicating the presence of enhanced lipid peroxidation as a consequence of irradiation injury. Since physostigmine treatment prevented elevation in ileum MPO activity, ACh may serve to ameliorate radiation-induced oxidative injury.

IL-1*βa* and TNF-*α* increased in rat ileal mucosa and muscularis layers after a 10-Gy irradiation of the abdomen [44]. In radiation-induced proctitis, the levels of IL-1*β*, IL-2, IL-6 and IL-8 increase [45], and the expression of IL-10 and TNF-*α*, and apoptotic cell numbers, change in a time-specific and radiation-dose-specific manner [23]. The molecular pathogenesis of hepatocellular damage after irradiation is obscure. Cytokines are important for hepatocellular damage and repair and fibrosis development in other toxic liver injuries [37–39, 46]. Similarly, cell types from a range of organs interact by way of cytokines in the development of normal tissue reactions after radiation therapy [47]. The expression of the pro-inflammatory cytokines IL-1*β*, IL-6 and TNF-*α* was also upregulated after liver irradiation *in vivo* [48]. It was also demonstrated that irradiation increased the susceptibility of hepatocytes to TNF-*α*-mediated apoptosis through cell–cell interaction, leading to liver fibrosis [49]. It has also been shown that ACh, through activation of the muscarinic receptor, possesses an anti-apoptotic effect by increasing the expression of survival signaling molecules as well as activating the anti-oxidant systems. ACh inhibits long-term hypoxia-induced apoptosis by suppressing oxidative stress-mediated MAPK activation as well as regulation of Bcl-2, c-IAPs and caspase-3 in mouse embryonic stem cells [50]. Recently, it has been shown that ACh prevents angiotensin II-induced apoptosis in H9c2 cells through downregulation of the AT1 receptor and inhibition of reactive oxygen species-mediated p38 MAPK activation as well as regulation of Bcl-2, Bax and caspase-3 [51]. Apoptosis involves an organized series of biochemical events leading to the development of characteristic morphological traits including cell shrinkage, loss of cell-to-cell contact, and chromatin condensation [52]. The key event in the initiation of apoptosis is the activation of caspases [53]. In mammalian cells, caspase activation can be initiated by two distinct mechanisms: one is cell surface receptor-mediated [54] and the other is mediated by mitochondrial factors [55]. In each case, activation of a proximal caspase results in a cascade wherein downstream caspases are activated, culminating in the activation of terminal caspases such as caspase-3 that cleave structural and metabolic cellular proteins and result in cell death [56].

Radiation enteritis creates difficulty for patients receiving ionizing radiation directed to the abdomen or pelvis. Adjacent healthy tissues can also be affected, although the radiation is directed against the malignant tissue. Not only small intestine, but also hepatic and pancreatic toxicity may develop following irradiation. Our results demonstrated that physostigmine treatment protected the rat liver from the hazardous effects of irradiation since we observed well-preserved liver parenchyma with sinusoids, hepatocytes and Kupffer cells in both phases of irradiation. However, in a previous study performed with glutamine, no good results were obtained for liver in terms of myeloperoxidase activities or of malondialdehyde levels [57]. But melatonine pretreatment before irradiation was shown to be protective for liver, as evidenced by decreased malondialdehyde and increased glutathion levels [58]. The protective role of the cholinergic system in the liver should be investigated in future studies.

## CONCLUSION

In conclusion, our results may indicate that ACh has radio-protective effects by affecting the balance between cytokines and their antioxidant properties. Physostigmine, rather than atropine, treatment may be a possible adjuvant for use during radiotherapy.

## FUNDING

Funding to pay Open Access publication charges for this article was provided by the authors.
